# Why Do We Lose Bone During Weight Loss: Can It Be Prevented?

**DOI:** 10.1007/s11914-026-00984-z

**Published:** 2026-09-09

**Authors:** Sue A. Shapses, Brandon D. McGuire

**Affiliations:** 1 Department of Nutritional Sciences, New Jersey Institute of Food, Nutrition, and Health, Rutgers University, 59 Dudley Road, New Brunswick, NJ 08901 USA; 2 Department of Medicine, Rutgers-Robert Wood Johnson Medical School, New Brunswick, NJ 08901 USA

**Keywords:** Calcium absorption, Diet, Endocrine, Obesity, Weight loss

## Abstract

**Purpose of Review:**

Weight loss is recommended for obesity management, but it also increases bone turnover and reduces bone mineral density (BMD) and bone quality (microarchitecture, geometry, and strength). This review examines mechanisms and the multiple factors that influence skeletal responses to caloric restriction.

**Recent Findings:**

Bone loss during weight loss likely reflects multiple interacting mechanisms, including alterations in endocrine signaling, impaired calcium absorption, reduced mechanical loading, and alterations in the gut microbiota. The extent of bone loss is further influenced by age, sex, initial body weight, nutrient intake, and method of weight loss, with greater loss in older and lean adults, and after severe caloric restriction, such as with incretin mimetics or bariatric surgery. Current evidence emphasizes the importance of adequate nutrient intake (e.g., calcium, protein, etc.) and exercise as countermeasures to attenuate bone loss. Emerging evidence also suggests that additional countermeasures, including osteoporosis medications and other lifestyle interventions, may affect weight-loss-induced bone loss, and randomized trials are needed.

**Summary:**

Weight-loss-induced bone loss is multifactorial, involving endocrine, metabolic, nutritional, mechanical, and gut microbiota-mediated mechanisms that remain incompletely understood. These mechanisms influencing skeletal responses to weight loss can inform targeted strategies to preserve bone health and reduce fracture risk during obesity management.

Obesity is a complex chronic disease characterized by excess adiposity that increases the risk of obesity-related comorbidities and can negatively affect skeletal health. Individuals with obesity typically have greater bone mineral density (BMD) compared to age- and sex-matched individuals with a normal body mass index (BMI) [1, 2]. However, in humans, BMD and bone quality (microarchitecture, geometry, and strength) are lower relative to body mass in individuals with obesity [2, 3], suggesting diminished skeletal benefits of increased body mass. Similarly, high-fat diet-induced obesity in rodents attenuates the expected increase in bone mass and bone strength associated with greater body mass [4], with some studies also reporting a deterioration of bone quality [5]. The detrimental effect of a high-fat diet-induced obesity model on bone is possibly through excess saturated, but not monounsaturated fatty acid intake [6, 7], which can increase the expression of pro-inflammatory cytokines, including tumor necrosis factor-α and interleukin (IL)-1β and IL-6 [8]. Moreover, visceral adiposity may be especially harmful to BMD [9], and clinical data show that BMI-discordant abdominal thickness is a FRAX-independent risk factor for major osteoporotic fractures [10].

The loss of bone is multifactorial and can be driven by alterations in hormones, inflammation, nutrient intake, and mechanical loading. Although calorie-restricted (CR) diets are commonly encouraged for obesity management, the resulting weight loss can exacerbate these mechanisms, causing a reduction in BMD and increased susceptibility to frailty and fracture. The amount of weight-loss-induced bone loss associated with CR can be influenced by many factors, including age, sex, method of weight loss, and initial weight. In this review, we provide insight into current evidence on the effects of weight loss on BMD, bone quality, and fracture risk, and evaluate modifiable factors that may attenuate bone loss during CR.

## Weight Reduction

Voluntary weight loss of about 5–10% increases bone resorption [11] and is associated with 1-2.5% loss of total hip BMD [12, 13]. Following *≥* 1 year of moderate weight loss, reductions of approximately 5–9% in volumetric BMD and 2% in cortical thickness are reported [14, 15], and long-term weight loss (over 6–7 years) is associated with the deterioration of both cortical and trabecular microarchitecture in older adults [16, 17]. Weight loss may also decrease the trabecular bone score and it can increase the detection of vertebral abnormalities; these abnormalities may obfuscate the loss of lumbar spine BMD although affected vertebrae can be excluded to minimize this effect [18]. The loss of cortical bone and trabecular bone in energy restricted rodent models [19, 20] may be partly related to the increase in bone marrow adipose tissue (BMAT) that often accompany weight loss [20–22]. However, the change in BMAT is less clear in humans, as BMAT tends to decrease following diet-induced weight loss, but more variable responses are reported following bariatric surgery, possibly related to differences in study design, including the type of surgical intervention examined [23]. Cortical bone loss compromises bone strength by reducing failure load and increasing fracture susceptibility [24]. It has been estimated that there is a 3.5-fold increase in hip fractures in overweight and obese adults with a femoral BMD loss of 0.12 g/cm^2^ [13]. In the Look AHEAD study, weight loss exceeding 7% was associated with a 39% increased risk of frailty fractures, but not hip fractures [25].

In contrast to voluntary weight loss, involuntary weight loss may be more concerning for bone health, as underlying conditions, including gastrointestinal disorders, can compound the deleterious effects of weight loss on bone [26]. Additional factors, including frailty, immobilization, sarcopenia, sedentary behaviors, and medications, can further accelerate the rate of bone loss, especially in older adults [27, 28].

## Weight Cycling

The repeated cycle of weight loss and weight regain may cause cumulative damage to bone and increase the risk for fracture [29]. indeed, studies report that bone mass is not recovered with weight regain following 18–24 months after weight loss in older adults [30, 31]. moreover, in premenopausal women, lower bone density has been reported among those with a history of weight cycling [32]. this is also supported by a rodent study, which showed reductions in bone quality and strength after weight cycling [33] providing further evidence of potential adverse skeletal effects.

## Non-Modifiable Risk Factors

The extent of bone loss accompanying weight reduction varies by age, menopausal status, and sex. In controlled weight loss trials in postmenopausal women, a 5–10% reduction in body weight generally results in a 1–2% decrease in BMD at the hip, femoral neck, and trochanter [13, 14, 34]. Older women and men (*≥* 65 years) typically exhibit similar weight-reduction-induced bone loss [15, 34, 35]. In addition, weight loss in perimenopausal women has also been shown to induce hip bone loss [36]. However, in younger premenopausal women or middle-aged men with overweight or obesity, moderate weight loss is not shown to result in bone loss, which may be partially attributed to higher levels of sex hormones and/or a greater amount of skeletal muscle compared to older adults [37–39]. The adverse effects of CR on bone mass and strength are also evident in aged, but not younger mature rodents [40]. In human studies, reductions in lean mass and age-related sarcopenia may further compound the skeletal consequences of weight loss, increasing the risk of frailty and fractures [41, 42].

Initial body weight may influence the skeletal response to weight loss. Leaner women (< 60 kg) experience more rapid bone loss with ≥ 5% weight reduction than heavier women (> 70 kg) [28]. Consistent with this observation, adults without obesity (~ 40 years; BMI: 25 kg/m^2^) in the CALERIE trial experienced BMD loss at the lumbar spine (-1.3%), hip (-1.7%), and femoral neck (-1.8%) after 24 months with moderate (~ 10%) weight loss [43], unlike similar aged adults with obesity [37]. Similarly, CR in lean rats compared to obese rats is more detrimental to bone [44]. Collectively, these findings suggest that advancing age, sex, and lower baseline body weight are important determinants of skeletal vulnerability during weight loss.

## Mechanism Regulating Bone During Caloric Restriction

Several mechanisms may explain the adverse skeletal effects of weight loss, including changes in calcium absorption, endocrine factors, mechanical loading, and the gut microbiota.

## Calcium Absorption and 25-hydroxyvitamin D

Caloric restriction reduces Ca absorption efficiency [45, 46], and endocrine changes that accompany CR may mediate this decline. For example, reductions in estradiol concentrations reduce Ca absorption [44, 45], and estradiol replacement in ovariectomized rats prevents this decline [47]. Increases in cortisol may also impair Ca absorption [46], and have been linked to bone loss during short-term CR [48, 49]. Serum PTH increases during weight loss [11, 45], and may represent a compensatory response to reduced Ca balance. In addition, intestinal Ca absorption declines with age, which may explain the more pronounced negative Ca balance in older adults compared to younger adults [50]. The rise in 25-hydroxyvitamin D [25(OH)D] due to weight loss is attributed to its release from adipose tissue [51] and/or changes in hepatic vitamin D 25-hydroxylase [52]. Since serum 25(OH)D rises proportionally with weight loss [48, 53, 54], it should not contribute to the weight-loss-induced reduction in Ca absorption or balance or decrease in BMD.

## Other Endocrine And Paracrine Factors

Insulin-like growth factor-1 (IGF-1) promotes osteoblast differentiation and increases BMD, and circulating levels decrease with CR in animals, contributing to impaired bone remodeling and cortical bone loss [55]. In humans, IGF-1 does not consistently decline with weight loss and instead appears to be associated with low protein intake [14, 56]. Weight loss also alters sex steroid signaling by reducing aromatization and increasing sex hormone-binding globulin, thereby reducing circulating estradiol [57, 58], which can promote a pro-inflammatory milieu that stimulates osteoclast activity and bone loss in older men and women [59, 60].

**Fig. 1:**
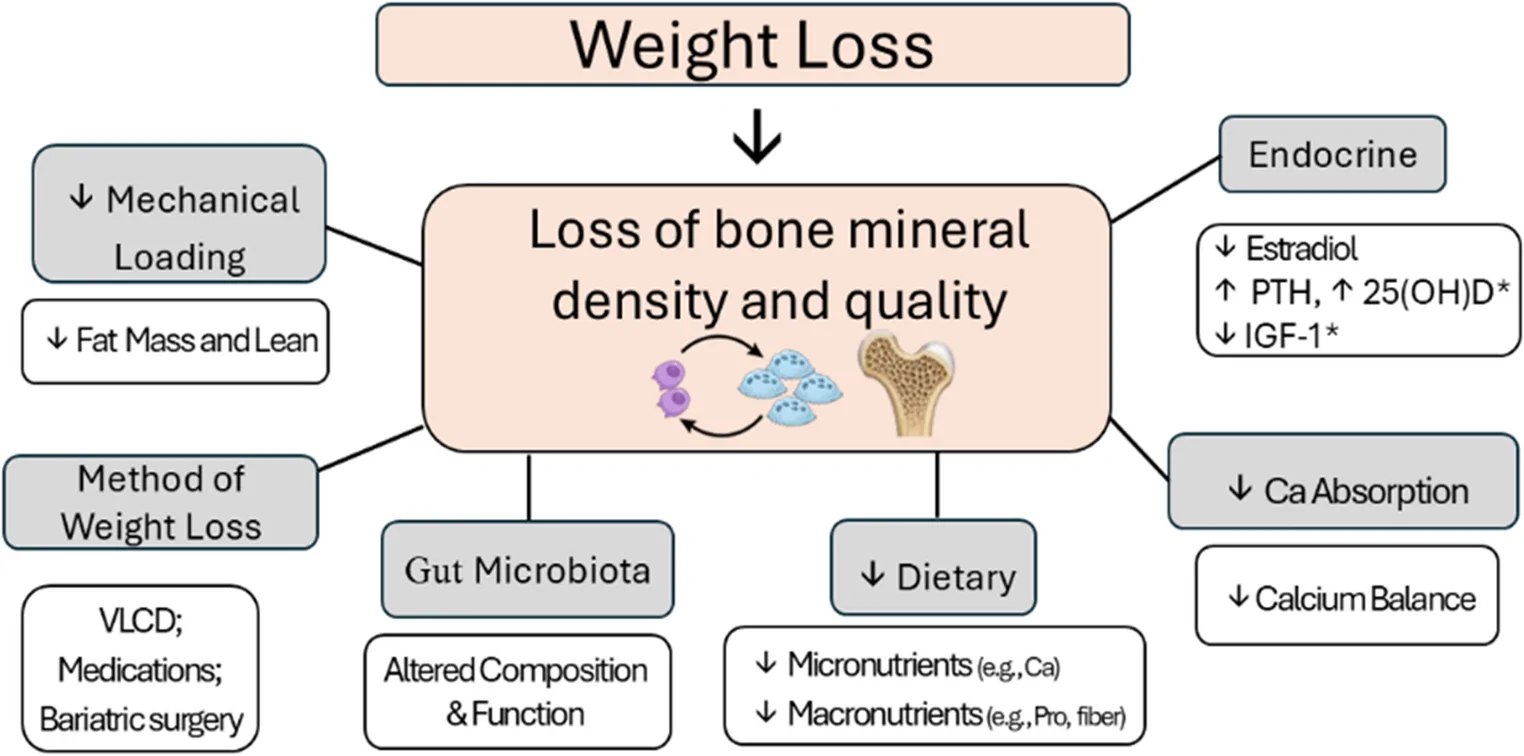
Factors influencing the loss of bone mineral density and quality with weight loss

Weight loss may also alter endocrine and paracrine signaling involving bone-derived, muscle-derived, and gut-derived factors. The osteokine, sclerostin, that inhibits Wnt/β-catenin signaling, increases with weight loss [58, 61]. Following weight loss, sclerostin is negatively associated with hip geometry parameters; however, exercise may attenuate these effects [61]. Irisin, a myokine, promotes osteoblastogenesis and increases bone mass [62]. Although irisin levels decrease with extreme weight loss [63], the implications for skeletal health remain unclear. Gut-derived peptides such as ghrelin, an orexigenic gut peptide, promote osteoblast differentiation and increase BMD [64]. Ghrelin increases with moderate weight loss, but declines following bariatric surgery, and this decline is associated with elevated bone resorption (C-telopeptide of type I collagen) and reduced BMD [58]. Other gut peptides, including glucose-dependent insulinotropic polypeptide and glucagon-like peptide 1 (GLP-1), also regulate bone remodeling and can be altered with weight loss, and are discussed in more detail below Fig. 1.

Alterations in adipokine signaling due to loss of adipose tissue with CR may also influence bone metabolism. The skeletal effects of leptin appear to be context-dependent, varying by central versus peripheral actions and skeletal site [65], and leptin has been shown to predict reductions in hip BMD during weight loss [66]. However, leptin administration during weight loss does not prevent BMD loss [67]. Adiponectin, an anti-inflammatory adipokine, increases with CR [68]. While it promotes osteoblastogenesis, and suppresses osteoclast activity; it’s rise following weight loss is associated with lower BMD [69]. Other adipokines, including adipsin [70], are also altered with weight reduction, but overall, the relationship between all adipokines and bone following weight loss remains unclear [71].

## Gut Microbiome

Caloric restriction alters the gut microbiota composition, intestinal permeability, inflammation, and microbial-derived metabolites. A meta-analysis of 47 intentional weight-loss trials (*n* = 1916; 81% female) reported increased alpha-diversity and reduced intestinal permeability after 6 months [72]. Similarly, CR studies often report enrichment of SCFA-producing microbes, including *Akkermansia* and *Bifidobacterium*, alongside reductions in pro-inflammatory taxa [73]. These changes provide a plausible link to skeletal health [74]. Following extreme weight loss with bariatric surgery, alterations in the gut microbiota are associated with femoral neck BMD loss, potentially related to lower SCFA concentrations [75]. Dietary fiber [76] and probiotics [77] may further optimize weight-loss-induced microbiota changes to support skeletal health.

## Mechanical Loading

Mechanical loading is a driver of bone formation [78]. With weight reduction, a natural unloading of the bone can occur, which can suppress osteoblastic activity and promote bone resorption [78]. Although some propose that this response is a physiological adaptation to reduced body size, others argue it represents a pathological overshoot that increases skeletal fragility [79]. In all likelihood, weight-loss-induced bone loss involves both physiological and pathological responses, with skeletal adaptations exceeding those expected for the reduced body size [79]. Moreover, weight-loss-induced bone loss occurs at both weight-bearing and non-weight-bearing sites [14], can continue despite the cessation of CR, and is not recovered in individuals who regain weight [30, 31].

## Weight Loss Method, Magnitude, And Rate

The amount and rate of weight loss are greater with very low-calorie diets (vlcd), bariatric surgery, or with incretin medications compared to lifestyle interventions.

## Very Low-Calorie Diets

VLCDs are typically defined as ≤800 kcal/day and consist of total or partial meal replacement with shakes or other nutrient-dense foods and are typically ≤ 4 months in duration. In the largest trial conducted[80], postmenopausal women with obesity were randomized to either moderate CR (25-30% reduced calories) or to severe CR (65% reduced calories) for 4 months (or until BMI of 20 kg/m^2^ was achieved), followed by moderate CR for 8 more months [80]. Weight loss was 15 kg and 8 kg in the severe and moderate CR groups, respectively. Participants randomized to severe CR experienced 1.5-fold greater lean mass loss and 2.5-fold greater total hip BMD loss than those assigned to moderate CR. Notably, greater hip bone loss persisted after adjustment for body weight, suggesting that the rate of weight loss, in addition to the amount, contributes to bone loss.

## Bariatric Surgery

Bariatric surgery is another form of severe weight loss for patients with severe obesity (BMI > 35 kg/m 2 ). The most common of these procedures are the sleeve gastrectomy and the Roux-en-Y gastric bypass, and result in a total weight loss of 15–30% with some malabsorption. Both the amount of weight loss and the extent of malabsorption affect bone loss after bariatric surgery. In adults and adolescents, there is well-documented bone loss [81, 82], compromised bone strength and microstructure [83, 84], and increased risk of fracture [85]. The decline in Ca absorption after gastric bypass surgery likely contributes to secondary hyperparathyroidism [86, 87], whereas the chronically higher parathyroid hormone (PTH) levels can explain a greater cortical bone loss [88]. Unfortunately, vitamin D supplementation (2000 IU/d or 3500 IU/d or greater) in bariatric patients that increases serum 25(OH)D has little to no effect on PTH [89]. In addition, vitamin D does not reduce bone turnover or prevent bone loss in bariatric patients [86, 90, 91]. Also, while large changes in soft tissue can introduce DXA measurement error, especially at the lumbar spine, this error may primarily affect measurements following extreme weight loss and is unlikely to affect measurements following moderate weight loss [92].

## GLP-1 Receptor Agonists

The incretin mimetic, GLP-1 receptor agonists (GLP-1RA) stimulate insulin secretion, reduce glucagon secretion, slow gastric emptying, and promote satiety. For nearly two decades, GLP-1RA’s have been used to improve glycemic control in patients with diabetes. more recently, higher doses of incretin based therapies have demonstrated clinically significant weight loss (13-22%) [93–95]. Moreover, a triple agonist medication with glucagon (i.e., retatrutide) resulted in up to 24% weight loss [96], and all GLP-1RA medications show significant health benefits. however, there is also some concern that GLP-1-RA-based therapy may contribute to excessive loss of lean mass [97, 98]. The effect on bone is less well known. animal studies indicate positive effects of incretins by reducing osteoclastogenesis, bone turnover, and BMD [99]. In human trials, early studies in patients with type 2 diabetes indicate that GLP-1RAs increase bone resorption, but do not change BMD at the femoral neck, the total hip, and the lumbar spine [100] or exacerbate the risk of bone fragility [101]. In a small phase 2 trial, semaglutide increased bone resorption and reduced BMD with 9% weight loss compared to placebo (no weight loss) over one year in older adults with low bone mass [102]. this study does not show a specific detrimental effect of semaglutide since the control group did not lose weight. moreover, in a well controlled one-year trial following an 8-week VLCD (12% weight loss), adults with obesity (18–65 years of age) were randomized to placebo, a moderate- to vigorous-intensity exercise program, liraglutide, or the combination of exercise and liraglutide [103]. Although the exercise and liraglutide group achieved the greatest weight loss (16%), hip and lumbar spine BMD were not greater than the placebo group (7%). similar to weight loss without liraglutide, there is a beneficial role of exercise on bone during CR [15, 66]. Also, while the liraglutide alone group had greater hip BMD loss (0.026 g/cm2) compared to placebo (0.012 g/cm2), there was also aproportionally greater weight loss (14% weight loss) compared to placebo (7% weight loss). Currently, the amount of bone loss observed with incretin-based therapies may be largely explained by the greater extent and rate of weight loss compared to lifestyle-induced weight loss. Lastly, the effect of GLP-1RA medications on fracture risk remains unclear. Most available evidence examines diabetic patients with or without obesity and suggests a neutral or potentially protective effect; however, evidence in individuals using these medications for obesity treatment remains limited [104].

## Countermeasures to Prevent Bone Loss 

### Micronutrients

Adequate Ca intake is an important component for strategies to minimize bone loss during intentional weight reduction. Weight loss increases bone turnover and can reduce BMD, particularly in postmenopausal women and older adults. This is especially concerning during CR, as Ca consumption decreases to levels below recommended intakes (e.g., ~ 650 mg/day) [48, 105] and intestinal Ca absorption is reduced in older women [45]. During CR, net Ca absorption is 140 mg/day, which is below the 200 mg absorbed Ca/day estimated to offset obligatory Ca loss [45, 54, 106]. This 60 mg Ca/day deficit is estimated to cause an additional annual bone loss of approximately 0.9% [54]. During CR, postmenopausal women with overweight who were randomized to higher Ca intake from diet and supplement at 1.7 g/d compared to 1 g Ca/day had reduced bone turnover and attenuated BMD loss [105]. Others showed that 1 g/d supplemental Ca intake attenuates weight-loss-induced bone loss in women with obesity [107].

Serum 25(OH)D concentrations, which are lower with obesity, rise after CR loss [48, 53, 54]. In addition, a higher dose of vitamin D (2500 IU/d) during CR increases intestinal Ca absorption, but a positive Ca balance can be achieved even at a vitamin D dose of 600 IU/day as long as there is adequate Ca intake (1.2 g/day) [54]. Higher doses of vitamin D (2000–4000 IU/d) have been examined over one year in postmenopausal women [108, 109] showing no differential effect on BMD (hip, femoral neck, or spine) or bone quality. While higher doses of vitamin D intake may not further improve outcomes, adequate intake is important, especially during winter dieting due to seasonal lower 25(OH)D levels [37]. Other micronutrients are often reduced during CR, which could negatively influence bone mass, and strategies to ensure adequate intake, such as a multivitamin/mineral supplement, should be considered.

### Macronutrients

The proportional reduction in all macronutrients to achieve a well-balanced CR diet can influence outcomes and also be a challenge for successful weight reduction. Very low carbohydrate intakes, such as those associated with ketogenic diets can contribute to bone loss [110, 111]. In an 8-week inpatient metabolic study, healthy participants consumed a very low-carbohydrate diet that was high in fat and protein [111]. The diet markedly increased renal acid load and urinary Ca excretion without increasing intestinal Ca absorption, ultimately decreasing estimated Ca balance [111]. Ketogenic diets, used to effectively treat children with epilepsy, have also been associated with progressive bone loss [110]. However, a systematic review concluded that there remains limited evidence that ketogenic diets adversely affect bone health independent of weight loss [112]. Also, moderate weight loss with a low-carbohydrate diet, without ketosis, show no differential effect on BMD over one to two years [38, 113].

Protein intake can increase the acid load [114] and urinary Ca excretion [115]. This had led to concerns that a higher protein intake could be harmful to bone. However, a higher protein intake also increases intestinal Ca absorption [116]. This is consistent with high protein intake studies (e.g., 2.4–2.7 g/kg/d) showing no increase in short-term bone resorption [117] or disruption of Ca homeostasis or balance [118]. A meta-analysis including 16 RCTs and 20 prospective cohort studies examining the effects of protein on bone reported a significant attenuation of BMD loss at the lumbar spine and trends toward mitigation of BMD loss at other bone sites [119]. In a recent 18-month study (6 months of weight loss and 12 months of weight maintenance) in 187 older adults (72 years) showed that a higher protein intake (1.2 g/kg/d) compared to recommended intake (0.8 g/kg/d) had a beneficial effect on hip bone strength at 6 months, but not 18 months, with 5–8% weight reduction [120]. In addition, protein source (e.g., animal vs. plant) is currently not shown to differentially affect BMD [119].

Specific fatty acids (e.g., saturated, polyunsaturated, and monounsaturated) affect bone [7, 121] but has not been studied during weight loss. Food components (e.g., fiber, polyphenols, probiotics, antioxidants) and dietary patterns also influence bone and could be addressed in future studies during caloric restriction.

### Physical Activity

Mechanical loading induced by exercise can attenuate BMD loss during weight loss [15, 34, 66, 122], although this effect may be site-specific and varies by study, possibly due to differences in study duration, exercise modality, and intensity [123]. Consistent with the role of mechanical loading, it has been hypothesized that replacing external load with a weighted vest may attenuate weight-loss-induced bone loss. However, a large RCT reported similar hip bone loss in participants with or without weighted vests during weight loss [124], although secondary analysis suggests that the beneficial effect of a weighted vest may be influenced by the time spent standing [125].

### Intermittent Fasting And Circadian Rhythms

Intermittent fasting protocols are used for weight loss, and feeding-fasting rhythms alter circadian clocks throughout the body, including those in osteoblasts and osteoclasts [126]. Circadian disruption impairs bone remodeling by altering osteoblast differentiation and receptor activator of nuclear factor-kappa B ligand (RANKL) signaling, increasing bone resorption, and reducing bone mass and microarchitecture in knockout and dietary-induced disruption models [127, 128]. Preclinical findings suggest that dietary fasting interventions have context-dependent skeletal effects, protecting against inducible bone loss in middle-aged or older animals but impairing skeletal development in young animals, likely related to spontaneous CR [129, 130]. In humans, fasting interventions, including TRE, are hypothesized to have modest bone-sparing effects; however, evidence remains limited [12]. Only one TRE study has evaluated clinically relevant fracture sites [131]. Unlike the CR comparison group (~ 6% weight loss), which experienced hip BMD loss, TRE did not result in significant weight loss or BMD loss in older adults [131]. Our preliminary data suggest that TRE + CR prevents radius BMD loss compared to CR alone and is influenced by changes to the microbiome [132]. Therefore, although preclinical evidence suggests that TRE attenuates inducible bone loss, additional human trials are needed.

### Osteoporosis Medications

Pharmacological treatment for osteoporosis may help attenuate bone loss associated with weight reduction. Early studies in postmenopausal women undergoing modest exercise-induced weight loss (~4 kg) demonstrated decreases in BMD at the lumbar spine, total hip, and trochanter, with no apparent benefit of raloxifene or estrogen therapy on skeletal outcomes [133]. A pilot study following sleeve gastrectomy showed that risedronate, compared with placebo, attenuates BMD loss at the hip and spine over 6 months [134]. A study from Japan has reported that bisphosphonates may attenuate involuntary age-related weight loss in addition to their established skeletal benefits [135]. Ongoing studies will provide further insight into the role of osteoporosis therapies during intentional weight reduction. In particular, the Bone, Exercise, Alendronate, and Caloric Restriction (BEACON) trial is evaluating the independent and combined effects of a 12-month intervention consisting of resistance and bone-loading exercise together with alendronate treatment on weight loss-associated bone loss in adults aged 60-years and older [136].

## Conclusion

Weight loss is recommended for obesity management; however, it can also cause bone loss, and the extent of this loss is influenced by multiple interacting mechanisms and individual characteristics. Mechanistically, alterations in endocrine signaling, Ca absorption, mechanical loading, and the gut microbiota can contribute to this loss of bone, and an individual’s age, sex, initial body weight, nutrient intake, and the method and rate of weight loss can modify its severity. Although adequate Ca and protein intake and exercise can attenuate weight-loss-induced bone loss, additional strategies are important given that effective obesity treatments produce greater weight reduction. As a result, the preservation of bone becomes even more important, especially in vulnerable populations. Further research should identify mechanisms regulating bone loss during CR and examine interventions to preserve bone, including novel dietary components, exercise, osteoporosis medications, and examine multimodal interventions to preserve skeletal health during weight loss.

## Key References

- Gregori G, Mediwala S, Liebschner M, Kim D, Bryant MS, Klonis N, et al. Bone quality response to lifestyle intervention in older adults with obesity (LIMB-Q trial): a randomised controlled trial. Lancet Healthy Longev. 2025;6(9):100761.
  - ○ This 12-month randomized controlled trial using high-resolution peripheral quantitative CT and finite element analysis showed that an intensive lifestyle intervention (weight management and exercise) preserved bone quality during weight loss compared to an educational control group in older adults with obesity.
- Beavers KM, Lynch SD, Fanning J, Howard M, Lawrence E, Lenchik L, et al. Weighted Vest Use or Resistance Exercise to Offset Weight Loss-Associated Bone Loss in Older Adults: A Randomized Clinical Trial. JAMA Netw Open. 2025;8(6):e2516772.
  - ○ The INVEST trial showed that replacing mechanical loading with daily use of a weighted vest did not attenuate the concomitant bone loss associated with weight loss over 12 months in older adults with obesity, although the effects may be moderated by time spent in the standing position [125].
- Seimon RV, Wild-Taylor AL, Keating SE, McClintock S, Harper C, Gibson AA, et al. Effect of Weight Loss via Severe vs Moderate Energy Restriction on Lean Mass and Body Composition Among Postmenopausal Women With Obesity: The TEMPO Diet Randomized Clinical Trial. JAMA Netw Open. 2019;2(10):e1913733.
  - ○ This randomized trial in postmenopausal women highlights the importance of weight-loss rate and its effect on bone mineral density, with severe energy restriction resulting in 2-fold greater weight loss and 2.5 times as much hip BMD loss compared to moderate energy restriction over 12 months.
- Zibellini J, Seimon RV, Lee CM, et al. Does Diet-Induced Weight Loss Lead to Bone Loss in Overweight or Obese Adults? A Systematic Review and Meta-Analysis of Clinical Trials. J Bone Miner Res. 2015;30(12):2168-78.
  - ○ This analysis with 41 publications included adults with overweight or obesity who followed a moderate weight-loss intervention and reported a reduction in total hip BMD.
- Cifuentes M, Riedt CS, Brolin RE, Field MP, Sherrell RM, Shapses SA. Weight loss and calcium intake influence calcium absorption in overweight postmenopausal women. Am J Clin Nutr. 2004;80(1):123-30.
  - ○ This study in postmenopausal women is important because it establishes that caloric restriction reduces Ca absorption, which can compromise Ca balance and contribute to bone loss.

## Data Availability

No datasets were generated or analysed during the current study.
